# Supplementary material for: Pulmonary Function and Associated Prognostic Factors in Children After COVID-19: A Retrospective Cohort Study
Source: Medicina (Kaunas). 2025 Nov 29;61(12):2136. doi: 10.3390/medicina61122136 (PMC12735147; doi:10.3390/medicina61122136)

Supplementary Table S1. Spirometry results based on parameters

Parameter	(Mean ± SD) / (Median; range)
FEV1/FVC	105.85 (100.2 – 110)
FEV1 (%)	77.9 (66.78 – 92.48)
FVC (%)	82.4 (67 – 90.1)
FEF50 (%)	111.87 ± 53.6
FEF25 (%)	116 (81.25 – 153.5)

Data are presented as mean ± standard deviation (SD) for normally distributed variables, and median (interquartile range) for non-normally distributed variable.

Supplementary Table S2. Bivariate analysis between persistent symptom and spirometry result

Variable	Spirometry; n (%)		*P*	RR (95% CI)
	Abnormal	Normal		
**Presence of persistent symptom**				
Yes	14 (77.8)	4 (22.2)	**0.03**	1.99 (1.38–2.87)
No	32 (39.0)	50 (61.0)		
**Number of persistent symptoms**				
> 1 symptom	11 (91.7)	1 (8.3)	0.08	1.83 (0.81–4.15)
1 symptom	3 (50.0)	3 (50.0)		

Supplementary Figure S1. Comparison between groups based on the number of symptoms complained (n)

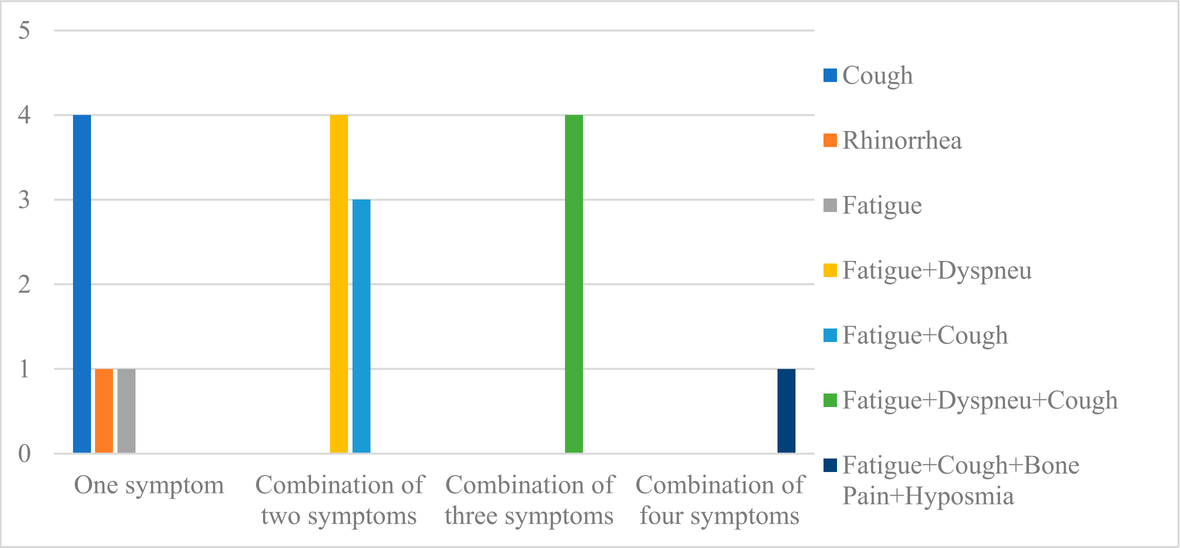

Supplement: Supplementary file 1 [file medicina-61-02136-s001.zip › medicina-3816187-supplementary.pdf]
